# The role of RNA modifications in hepatocellular carcinoma: functional mechanism and potential applications

**DOI:** 10.3389/fimmu.2024.1439485

**Published:** 2024-08-20

**Authors:** Jin-Xiu Liu, Xiaoping Zhang, Wen-Hua Xu, Xiao-Dan Hao

**Affiliations:** ^1^ Institute for Translational Medicine, The Affiliated Hospital of Qingdao University, College of Medicine, Qingdao University, Qingdao, China; ^2^ Department of Ophthalmology, The Affiliated Hospital of Qingdao University, Qingdao, Shandong, China; ^3^ Institute of Regenerative Medicine and Laboratory Technology Innovation, Qingdao University, Qingdao, Shandong, China

**Keywords:** hepatocellular carcinoma, N6-methyladenosine, 5-methylcytosine, N1-methyladenosine, N7-methylguanosine, biomarkers, therapeutic targets

## Abstract

Hepatocellular carcinoma (HCC) is a highly aggressive cancer with a poor prognosis. The molecular mechanisms underlying its development remain unclear. Recent studies have highlighted the crucial role of RNA modifications in HCC progression, which indicates their potential as therapeutic targets and biomarkers for managing HCC. In this review, we discuss the functional role and molecular mechanisms of RNA modifications in HCC through a review and summary of relevant literature, to explore the potential therapeutic agents and biomarkers for diagnostic and prognostic of HCC. This review indicates that specific RNA modification pathways, such as N6-methyladenosine, 5-methylcytosine, N7-methylguanosine, and N1-methyladenosine, are erroneously regulated and are involved in the proliferation, autophagy, innate immunity, invasion, metastasis, immune cell infiltration, and drug resistance of HCC. These findings provide a new perspective for understanding the molecular mechanisms of HCC, as well as potential targets for the diagnosis and treatment of HCC by targeting specific RNA-modifying enzymes or recognition proteins. More than ten RNA-modifying regulators showed the potential for use for the diagnosis, prognosis and treatment decision utility biomarkers of HCC. Their application value for HCC biomarkers necessitates extensive multi-center sample validation in the future. A growing number of RNA modifier inhibitors are being developed, but the lack of preclinical experiments and clinical studies targeting RNA modification in HCC poses a significant obstacle, and further research is needed to evaluate their application value in HCC treatment. In conclusion, this review provides an in-depth understanding of the complex interplay between RNA modifications and HCC while emphasizing the promising potential of RNA modifications as therapeutic targets and biomarkers for managing HCC.

## 1 Introduction

Liver cancer as one of the most prevalent malignant tumors worldwide, ranks as the sixth most frequently diagnosed cancer and the third leading cause of cancer-related deaths globally, with 865,000 new cases and 758,000 deaths in 2022, accounting for 4.3% and 7.8% of all malignant tumor morbidities and deaths respectively (1, 2). Hepatocellular carcinoma (HCC), as the most common liver cancer (75%–85% of cases) with the higher incidence and mortality rates, is the top three causes of cancer-related death in many countries (3, 4). HCC development is linked to multiple factors such as hepatitis virus infection, alcohol consumption, non-alcoholic fatty liver disease (NAFLD), and cirrhosis (2, 5). The primary risk factors for the development of HCC are infection with hepatitis B virus (HBV) and hepatitis C virus, with non-alcoholic steatohepatitis linked to metabolic syndrome or diabetes mellitus emerging as a more common risk factor in Western countries (5). HBV is a DNA virus that undergoes a complex life cycle involving reverse transcription. Chronic infection with this virus is a leading cause of liver cancer and cirrhosis on a global scale (6). In addition to environmental factors, certain genetic factors, such as gene mutations of TERT promoter, TP53, CTNNB1, amplifications of VEGFA, etc. also play a role in the occurrence and development of HCC (7, 8). The molecular mechanisms underlying HCC differ based on the specific genotoxic factors and causes (5). Despite advancements in our comprehension of the disease’s pathophysiology and triggers, this information has not yet been sufficient implemented in clinical settings.

Surgical therapies for HCC, including surgical resection and liver transplantation represent potentially curative options for appropriate candidates with tumors detected at earlier stages (4, 9). However, only a minority of patients are eligible for this treatment because of factors such as cirrhosis (10), and local ablation is the preferred method for patients diagnosed with HCC in its early stages who are not candidates for surgery or transplantation (4). For patients with intermediate-stage HCC, chemoembolization is the main treatment strategy. Due to the suboptimal sensitivity of existing HCC surveillance tools and their underutilization in clinical practice, most patients with HCC are diagnosed at an advanced stage, which leaves minimal options for effective treatment (4, 11). Despite the advancements in immune-checkpoint inhibitor-based therapies, the objective response rate for patients with advanced-stage HCC is only around 30%, and the 3-year overall survival rate is still below 50% (4). Challenges faced in the management of HCC include difficulties in early diagnosis, high rates of recurrence, poor prognosis, limited effective treatment options and drug resistance (12).

RNA modification refers to the process of chemically modifying RNA, which can impact RNA stability, translation efficiency, and function (13). Recently, there has been increasing interest in studying RNA modifications in HCC. RNA modification can promote HCC progression by regulating other risk factors for HCC, such as fat metabolism and virus life cycle. RNA modifications have the ability to control viral replication by either modifying the viral genome or altering the expression of genes crucial for viral replication (6). NAFLD stands as a significant risk factor for the development of HCC. Researchers have revealed the impact of RNA modifications on crucial aspects including steatosis, inflammation, fibrosis, and tumorigenesis. RNA modification induces NAFLD by regulating lipid metabolism, ultimately leading to HCC transformation (14). More and more evidences suggest that aberrant modifications of specific RNAs have been correlated with the occurrence, development, metastasis, and prognosis of HCC (15). One common RNA methylation is N6-methyladenine (m6A), which has been found to be associated with tumor proliferation, metastasis, and drug resistance in HCC (16). Additionally, other RNA modifications, such as 5-methylcytosine (m5C), N1-methyladenosine (m1A), and N7-methyladenosine (M7G), have also been reported to play a role in the development and progression of HCC (17). Currently, research on the role of RNA modification in HCC mainly focuses on m6A, m1A, m5C, and m7G. Recent studies have highlighted the crucial role of these RNA modifications in HCC progression, which indicates their potential as therapeutic targets and biomarkers for managing HCC.

In this review, we comprehensively summarize the functional roles, molecular mechanisms, and potential clinical applications of various RNA modifications in HCC. Clarifying the functional mechanism of RNA modifications and identifying new therapeutic targets in HCC will provide novel strategies for treatment. Additionally, RNA modifications also exhibit potential as biomarkers for the early diagnosis and prognostic evaluation of HCC, thereby improving diagnostic accuracy and patient survival rates. Overall, this review enhances our understanding of the role of RNA modifications in HCC and offers new perspectives on its diagnosis, prognosis, and treatment.

## 2 RNA modifications

As early as the 1950s, scientists discovered that there were some special chemical modifications in RNA molecules (18–22). With advancements in technology and in-depth research, various RNA modification types have been discovered (23). Methylations on RNA nucleotides, such as m6A, were the earliest modification type identified. In 1955, scientists first discovered methylations in RNA molecules (18). Subsequently, there has been continuous identification of new RNA modifications, including m5C, m1A, m7G, and pseudouridine (23). Increasing evidence shows that RNA modifications play complex regulatory roles in the cell and exert important effects on gene expression and cellular functions (24, 25). Recent studies indicate that specific RNA modifications, such as m6A, m5C, m1A, and m7G, through a series of modification regulatory proteins, affect the fate of RNA molecules such as precursor RNA processing, RNA splicing, stability, transport processes and translation, thereby regulating the expression of HCC-associated genes, and are involved in the progression of HCC.

### 2.1 N6-methyladenosine

**Figure 1:**
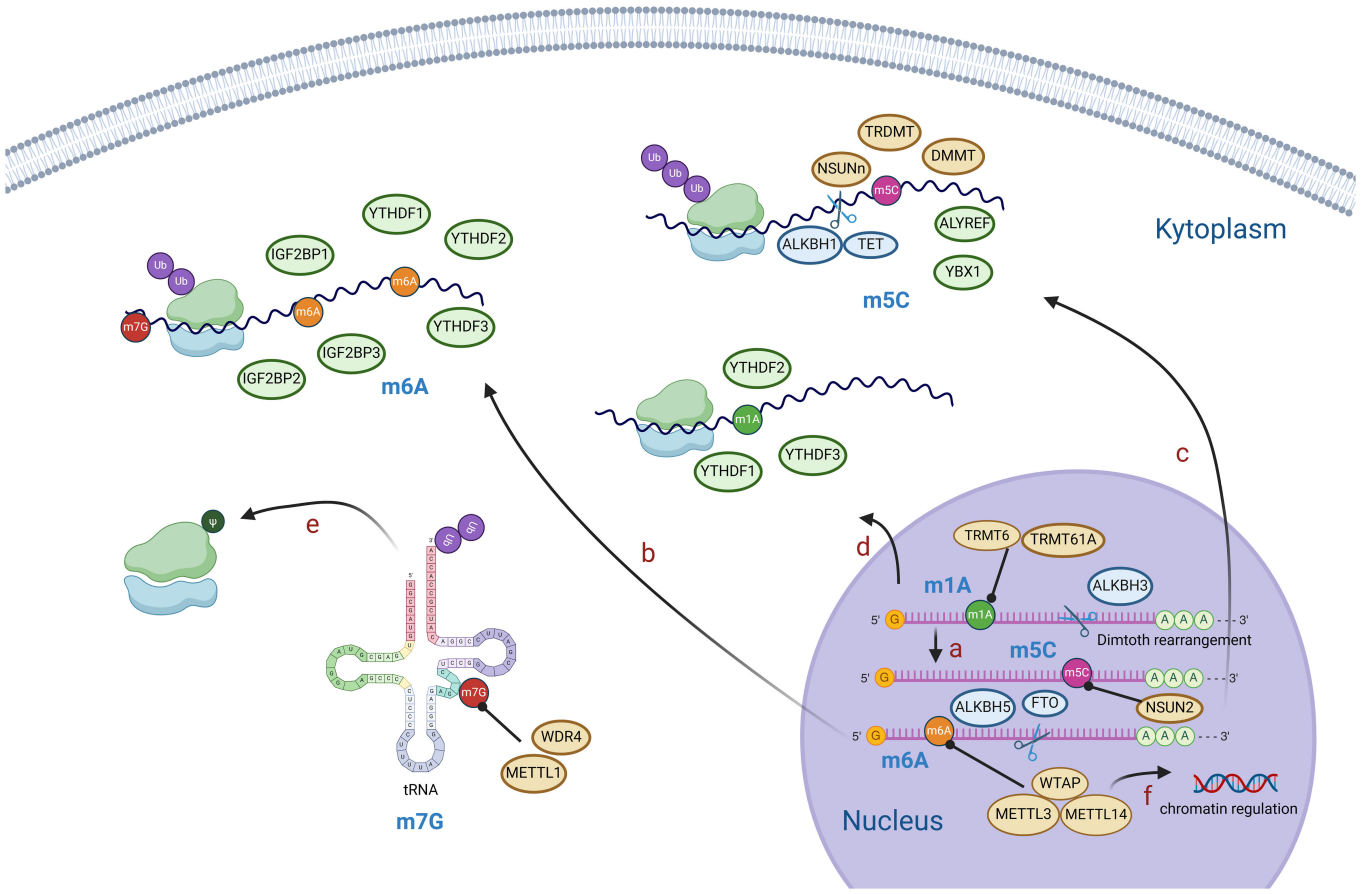
Diagram of RNA modification mechanisms. M1A is generally located in the 5’ end of mRNA and can be converted to m6A by Dimroth rearrangement **(A)**. M6A, m5C, m1A modifications mediate mRNA processing, stability of RNA, post-translational protein modification, etc **(B–D)**. M7G modification mainly affects tRNA and rRNA and mediates post-translational protein modification **(E)**. M6A modification of mRNA reversely regulates chromatin **(F)**. (Created with BioRender.com).

**Figure 2:**
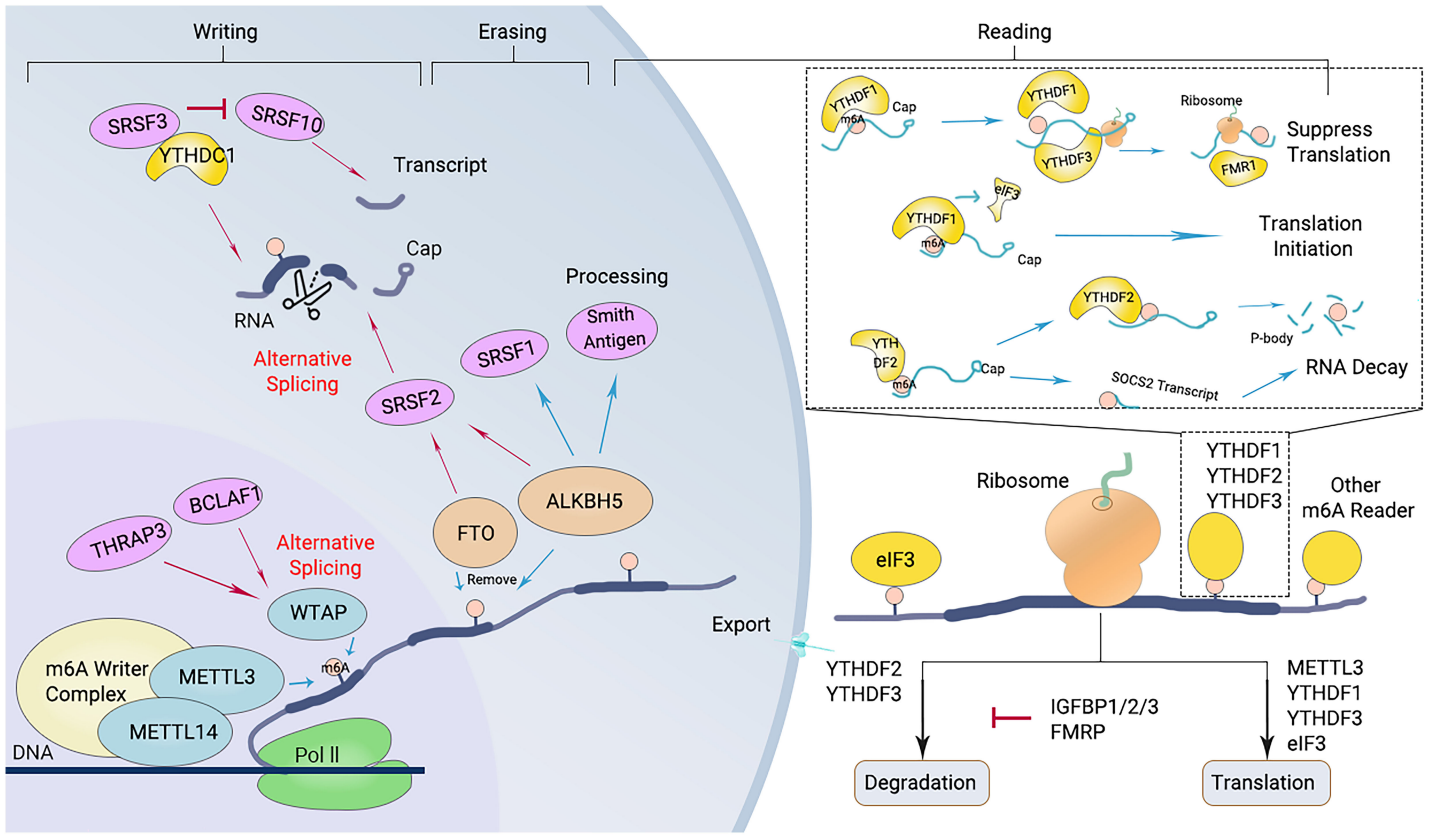
Diagram of the m6A modification mechanism. M6A “writers”, including METTL3/14 and WTAP, catalyze the m6A modification of adenosine on RNA. Removing the methylation of RNA needs the functions of m6A “erasers” that mainly consist of FTO and ALKBH5. M6A “readers” (such as YTHDF1/2/3 and others) recognize m6A modification sites and exert corresponding functions.

N6-methyladenosine, also referred to as m6A, is an RNA methylation that involves the transfer of a methyl group to adenosine (A) at N6 and is catalyzed by an RNA methyltransferase (**Figure 1**). It is the most prevalent, abundant, and evolutionarily conserved RNA methylation in eukaryotes (26). The regulation of RNA m6A methylation involves three groups of proteins: m6A methyltransferases (“writers”), m6A demethylases (“erasers”), and m6A methylation recognition proteins (“readers”) (**Figure 2**) (27). Accumulating evidence suggests that m6A is essential for the progression of HCC. The expression of m6A regulators, including “writer”, “eraser” and “reader” proteins, changed significantly in HCC (**Table 1**).

**Table 1:** The roles of m6A regulators in hepatocellular carcinoma.

M6A regulator	Change(regulator)	Target	Change (target)	Molecular Mechanism	Function	REF
Writer						
METTL3	Up	circ-CCT3	Up	circ-CCT3/miR-378a-3p/FLT1	Promotes HCC cell proliferation, invasion, and migration	(8)
	Up	circ-ARL3	Up	circ-ARL3/miR-1305 ceRNA	Promotes HCC progression	(28)
	Up	lncRNA GBAP1	Up	lncRNA GBAP1/miR-22-3p/BMPR1A/SMAD	Promotes the migration, invasion and proliferation of HCC cells	(29)
	Up	LINC00958	Up	LINC00958/miR-3619-5p/HDGF	Promotes the proliferation, migration and invasion of HCC	(30)
	Up	Lnc-CTHCC	Up	lnc-CTHCC/hnRNPK/YAP1	Promotes the growth and metastasis of HCC	(31)
	Up	LncRNA MAAS	Up	c-Myc/cyclins	Promotes the proliferation of HCC cells	(32)
	Up	PTEN	Up	HBV/METTL3/PTEN/IRF-3; PTEN/PI3K/AKT	Affects innate immunity and the development of HCC	(33)
	Up	ASPM	Up	Up-regulated ASPM expression	Promotes the proliferation, migration and invasion of HCC	(34)
	Up	EGFR	–	EGFR-pak2-erk5	Promotes drug resistance in HCC cells	(35)
	Up	MTF1	Up	Up-regulated MTF1 expression	Promotes tumor growth and migration of liver cancer	(36)
	Up	–		UBC9/SUMOylated METTL3/Snail	Promotes the growth and metastasis of HCC cells	(37)
METTL14	Down	circSTX6	Up	circSTX6/HNRNPD/ATF3	Accelerates HCC proliferation and tumorigenicity and strengthens tumor metastasis	(38)
	Down	USP48	Down	USP48/SIRT6	Stimulates HCC exacerbation	(39)
	Down	HNF3γ	Down	HNF3γ/OATP1B1/OATP1B3	Weakens the sorafenib response and promotes HCC progression	(40)
	Down	EGFR	Up	EGFR/PI3K/AKT	Promotes the migration, invasion, and EMT of HCC cells	(41)
METTL3/14	Down	ACLY; SCD1	Up	FA synthesis and lipid production	Leads to decreased HCC cell death and cell survival	(42)
WTAP	Up	ETS1	Up	WTAP/ETS1-p21/p27	Promotes the proliferative ability and tumor growth of HCC cells	(43)
	Up	LKB1	Down	WTAP/LKB1/AMPK	Resists autophagy and promotes cell proliferation	(44)
	Up	lncRNA AC115619	Down	lncRNA AC115619/WTAP	Promotes HCC progression	(45)
KIAA1429	Up	–	–	E-Ca/slug/snail	Promotes invasion, migration, and EMT of HCC	(46)
	Up	GATA3	Down	KIAA1429/GATA3	Promotes tumor growth and metastasis	(47)
Eraser						
ALKBH5	Down	LYPD1	Up	ALKBH5/LYPD1	Stimulates HCC exacerbation	(48)
	Down	LINC02551	Up	The ALKBH5/lnc C02551/DDX24 axis	Promotes HCC growth and metastasis	(49)
	Up	MAP3K8	Up	ALKBH5/MAP3K8; ERK/JNK/IL-8	Promote HCC cell proliferation and metastasis	(50)
	Up	HBX	Up	HBX/wdr5/h3k4me3	Inhibits the growth and migration of HBV-driven tumor cells	(51)
FTO	Up	GLUT1 and PKM2	Up	FTO-it1/FTO/c-Myc	Promotes HCC progression	(52)
	Down	GNAO1	Down	SIRT1/FTO/GNAO1	Enhances HCC proliferation and *in vitro* invasion	(53)
RALYL	Up	TGF-β2	Up	PI3K/AKT; STAT3 pathways	Promotes HCC tumorigenicity, self-renewal, chemoresistance, and metastasis	(54)
ZC3H13	Down	m6A modifications	Down	miR-362-3p/miR-425-5p-ZC3H13	Correlates with poor prognosis and poor outcome in HCC	(55)
Reader						
IGF2BP1	Up	circMDK	Up	miR-346/874-3p-ATG16L1; PI3K/AKT/mTOR	Promotes cancer cell proliferation, migration and invasion	(56)
	Up	circMAP3K4	Up	circMAP3K4-455aa/AIF	Prevents cisplatin-induced apoptosis of HCC cells	(57)
	Up	lncRNA MIR4435-2HG	Up	MIR44352HG/NOP58	Enhances the stem cell properties of HCC cells and promote tumorigenesis *in vitro* and *in vivo*	(58)
	Up	MGAT5	Up	Stability of MGAT5 mRNA	Promotes CSC liver phenotype and tumor metastasis	(59)
YTHDF1	Up	ATG2A and ATG14	Up	HIF-1α/YTHDF1/ATG2A/ATG14	Promotes hypoxia-induced autophagy of HCC and autophagy-related malignancies	(60)
YTHDF2	Up	OCT4	Up	m6A methylation of OCT4 mRNA	Promotes CSC liver phenotype and tumor metastasis	(61)
YTHDF3	Up	circ_KIAA1429	Up	TYHDF3/Zeb1/KIAA1429	Facilitates the migration, invasion, and EMT process of HCC	(62)
	Up	EGFR	Up	YTHDF3/m6A‐EGFR/STAT3 and EMT axis	Promotes the proliferation, invasion and migration of HCC cells	(63)
	Up	PFKL	Up	YTHDF3/m6A‐ PFKL	Promotes proliferation, migration and invasion of HCC cells	(64)
LRPPRC	Up	PD-L1	Up	Up-regulated PD-L1 expression	Promotes tumor growth, improve tumor immunity and immune infiltration	(65)

HCC, hepatocellular carcinoma; REF, references; EMT, epithelial-mesenchymal transition; CSC, cancer stem cell.

The “writers” refer to the m6A methyltransferases, which include methyltransferase-like protein (METTL) 3, METTL14, Wilms’ tumor 1-associating protein (WTAP), and vir like m6A methyltransferase associated (KIAA1429). These enzymes are responsible for catalyzing m6A modification (**Figure 2**) (27). The methylation of RNA to m6A is primarily carried out by the METTL3/METTL14 complex. Within this complex, METTL3 acts as the catalytic agent, while METTL14 serves as an allosteric activator that aids in binding to target RNA (46, 66).

The m6A modification of RNA is reversible, as it can be removed by “eraser” enzymes such as the m6A demethylases AlkB homolog 5 (ALKBH5) and fat mass and obesity-associated protein (FTO). These enzymes can convert m6A to A and rapidly remove m6A in a dynamic manner (**Figure 2**) (67). FTO belongs to the Alkb dioxygenase family and is associated with obesity (26). Knockdown of FTO significantly increases the level of RNA m6A modification (26). Another important demethylase, ALKBH5, is responsible for demethylating mRNAs within the nucleus. Knockout of ALKBH5 results in a significant increase in the level of RNA m6A modification (26, 67).

m6A “readers,” such as embryonic tumor-associated RNA-binding protein (IGF2BPs, insulin like growth factor 2 mRNA binding proteins), proteins containing YTH domains (YTH family proteins), and leucine-rich PPR-motif-containing protein (LRPPRC), have the ability to recognize and bind to m6A-modified RNA. Subsequently, they regulate the expression of related genes through various processes (**Figure 2**) (68). Proteins with YTH domains (YTHDF), such as YTHDF1, YTHDF2, and YTHDF3, can directly bind to m6A and then regulate translation or RNA decay. Specifically, YTHDF1 primarily enhances the translation efficiency of m6A-modified mRNAs, while YTHDF2 accelerates the degradation of m6A-modified mRNAs by recruiting several complexes to promote their degradation (61). The function of YTHDF3 is relatively complex, as it interacts with both YTHDF1 to promote protein synthesis and with YTHDF2 to facilitate mRNA degradation (69). IGF2BPs increase the stability and translational efficiency of m6A-modified mRNAs by recognizing m6A (70). Different members of the IGF2BP family may exhibit differences in regulating mRNA translation and stability; for example, IGF2BP1 and IGF2BP2 may have a wider range of substrate mRNAs in some cell types, whereas IGF2BP3 may focus more on specific mRNA molecules (71).

### 2.2 5-methylcytosine

5-Methylcytosine, also known as m5C, plays a pivotal role in the modification of RNA. It is produced during RNA synthesis by converting cytosine to 5-methylcytosine through the action of cytosine deaminase (**Figure 1**) (72). The methylation of RNA cytosines at the C5 position is facilitated by members of the NOL1/NOP2/SUN structural domain (NSUN) protein family and DNA (cytosine-5)-methyltransferase-like protein 2 (DNMT2). Additionally, this modification can be reversed by the AlkB homolog 1 (ALKBH1) and ten-eleven translocation (TET) demethylases (73). Furthermore, proteins such as Aly/REF export factor (ALYREF) and Y-Box binding protein 1 (YBX1) can recognize and bind to RNA m5C sites, resulting in downstream biological effects (74).m5C modification has been reported to regulate a broad variety of RNA functions, including increasing the stability of RNA molecules and increasing their stability in cells; affecting the translation efficiency of RNA; regulating the expression level of proteins by affecting the translation of mRNA and affecting the splicing process of RNA; and processing and trimming the precursor RNA, thus affecting its maturation and function (72, 75, 76). Via abnormal expression of regulators, m5C modification regulates the expression of HCC-associated genes, and is involved in the progression of HCC (**Table 2**).

**Table 2:** The role of other RNA modification regulators in hepatocellular carcinoma.

Category	Regulator	Change(regulator)	Target	Change (target)	Molecular Mechanism	Functions	REF
m5C							
Writer	USUN2	Up	LncRNA H19	Up	USUN2/H19RBA/G3BP1	Promotes the proliferation, migration and invasion of HCC cells	(77)
	USUN2	Up	GRB2	Up	Ras and p-Erk pathways	Promotes HCC progression and resistance to sorafenib	(78)
	NOP2	Down	XPD	Down	NOP2/XPD	Promotes the proliferation, migration, and invasion of HCC cells	(79)
Reader	ALYREF	Up	EGFR	Up	STAT3 signaling pathway	Promotes the progress of liver hepatocellular carcinoma	(80)
m1A							
Writer	TRMT6/TRMT61A	Up	tRNA	Up	Increases PPARδ translation	Triggers cholesterol synthesis, activates Hedgehog signaling, and drives self-renewal and tumorigenesis of hepatic CSC	(81)
Eraser	ALKBH3	Up	–	–	p21/p27-mediated cell-cycle arrest	Promotes HCC tumor cell proliferation and tumor formation	(82)
Reader	YTHDF1 YTHDF2 YTHDF3	Up	–	–	–	Regulates immune cell infiltration in HCC tissues	(83)
m7G							
Writer	WDR4	Up	CCNB1	Up	MYC/WDR4/CCNB1; PI3K/AKT; P53	Promotes the proliferation, metastasis, and sorafenib resistance of HCC	(84)
	WDR4	Up	TRIM28	Up	TRIM28/Target genes (IRF2, OCT4…)	Increases cell-acquired stemness and lenvatinib resistance	(85)
	METTL1	Up	tRNA	Up	m7G dependent translation control	Promotes hepatocarcinogenesis	(86)
	METTL1/WDR4	Up	tRNA	Up	EGFR pathway	Induces lenvatinib resistance in HCC cells	(87)
	METTL1/WDR4	Up	tRNA	Up	SLUG/SNAIL	Promotes the recurrence and metastasis of HCC after IRFA	(88)

HCC, hepatocellular carcinoma; REF, references; CSC, cancer stem cell.

### 2.3 N1-methyladenosine

N1-methyladenosine is a prevalent RNA modification primarily found on the adenylate residues of mRNAs. This modification is facilitated by an enzyme known as adenylate methyltransferase, which adds a methyl group to the guanine ribose ring of adenylate, resulting in m1A modification (89). The RNA m1A methyltransferases tRNA methyltransferase 10C (TRMT10C), 61B (TRMT61B), 6 (TRMT6), and 61A (TRMT61A) can be reversed by the ALKBH1 and AlkB homolog 3 (ALKBH3) demethylases (90). Additionally, YTHDF1, YTHDF2, YTHDF3, and YTH N6-methyladenosine RNA binding protein C1 (YTHDC1) serve as binding proteins that specifically recognize the m1A site and induce downstream effects (90). m1A modification plays a critical role in enhancing RNA stability, reducing the degradation rate (91), and protecting RNA molecules from damage in the external environment. This modification also extends their lifespan within the cell (92). Furthermore, m1A is involved in the regulation of RNA translation. Research has indicated that it can impact the efficiency and accuracy of RNA translation, thereby controlling protein synthesis levels (89). This may be achieved by influencing the assembly and recognition of translation initiation complexes (93). The abnormal expression of m1A regulators, including “writer”, “eraser” and “reader” proteins, were also found in HCC indicating a pivotal role of m1A in HCC (**Table 2**).

### 2.4 N7-methylguanosine

N7-methylguanosine, also referred to as m7G, is an RNA modification that involves the addition of a methyl group (-CH3) to the nitrogen atom at position 7 of the guanine nucleotide within the RNA molecule (88). This process is carried out by methyltransferase enzymes, such as METTL1 and WDR4 (WD repeat domain 4) (71). M7G modifications are primarily found in eukaryotic mRNAs and certain noncoding RNAs, including ribosomal RNA (rRNA) and transfer RNA (tRNA) (**Figure 1**) (94).

Similar to other modifications, m7G modification is also crucial for regulating gene expression. m7G modifications in RNA can help to stabilize mRNA molecules and reduce their degradation rate, thus extending the lifespan of mRNAs (95). In addition, m7G modification is involved in the translational regulation of mRNAs. The formation of a cap structure and m7G modification can affect the formation and recognition of translation initiation complexes, which in turn affects the rate and precision of protein synthesis (96). Furthermore, m7G modification also affects the transcriptional regulation of mRNAs, including steps such as splicing and translocation (94, 96). The upregulated expressions of m7G “writer” proteins WDR4 and METTL1, are associated with the progression of HCC via regulating the HCC-associated gene expressions (**Table 2**).

## 3 Functional mechanism of RNA modifications in HCC

### 3.1 m6A in HCC

**Figure 3:**
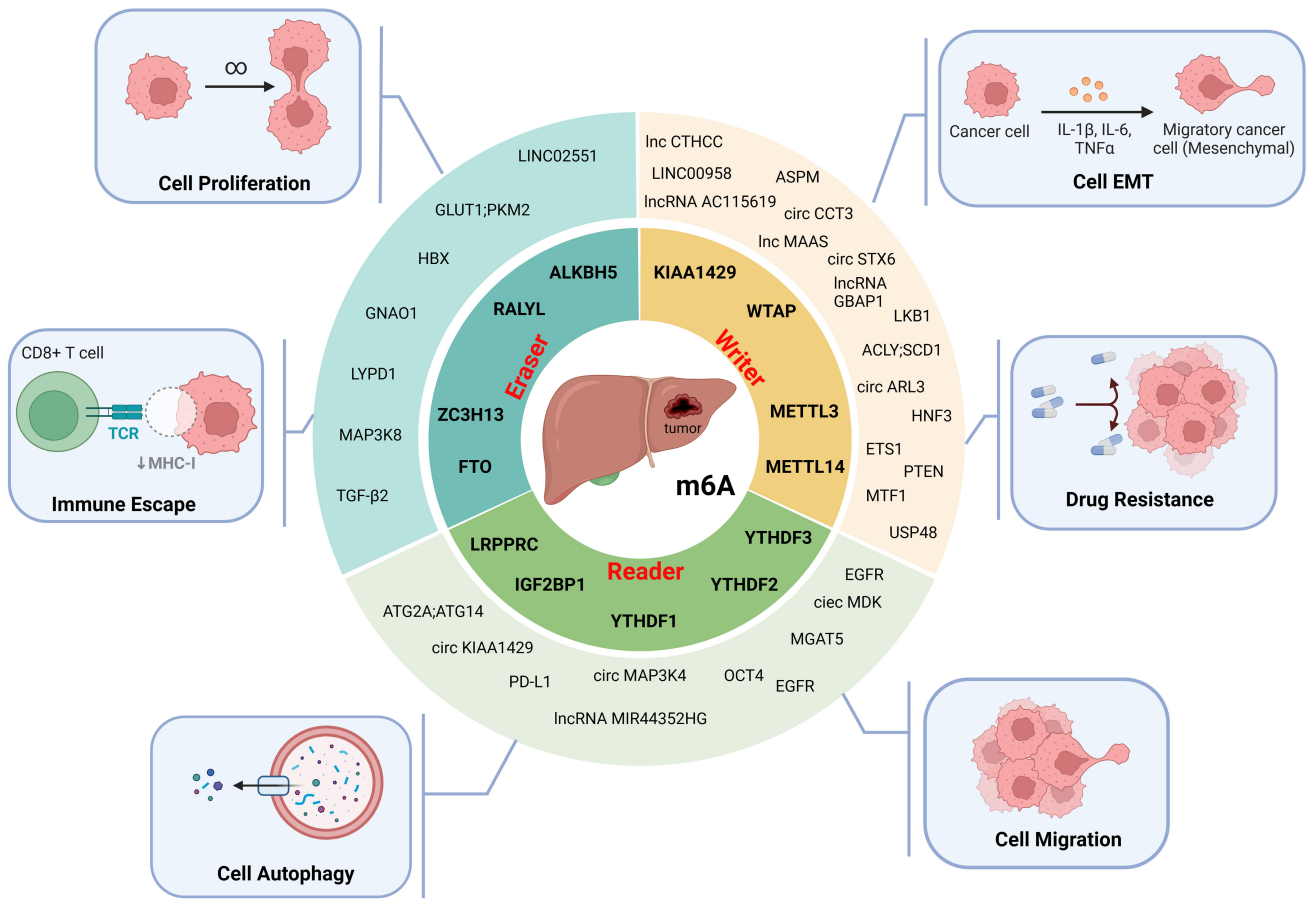
The molecular functions of m6A modification in HCC. M6A modifiers affect the proliferation, epithelial-mesenchymal transition (EMT), migration, autophagy, immune escape and drug resistance of HCC cells. (Created with BioRender.com).

By regulating the RNA m6A modification of HCC-associated genes, m6A regulators are involved in HCC cell proliferation, invasion, migration, epithelial–mesenchymal transition (EMT), autophagy, immune evasion, and drug resistance and play important roles in the progression of HCC (**Table 2**, **Figure 3**).

#### 3.1.1 The “writers” and HCC

##### 3.1.1.1 METTL3

METTL3, the first methyltransferase found to be involved in m6A modification, is significantly upregulated in HCC (29). By modifying the methylation of circular RNA (circRNA) (28, 66), long noncoding RNA (lncRNA) (29–32), and transcripts of other cancer-associated genes (33–37), METTL3 is involved in the progression of HCC by regulating the stability of related RNAs (**Figure 3**, **Table 1**).

Circ-CCT3 and circ-ARL3 play oncogenic roles in HCC. High expression of circ-CCT3 has been reported to be associated with poor prognosis in HCC patients (66). METTL3 can increase the level of m6A modification of circ-CCT3; promote HCC cell proliferation, invasion, and migration through the circ-CCT3/miR-378a-3p/FLT1 axis; and subsequently promote HCC progression (66). The upregulated expression of METTL3 caused by HBx (an X protein encoded by hepatitis B virus) increases the m6A modification level of circ-ARL3 and then leads to increased stability and enhanced expression of circ-ARL3, causing dysregulation of the circ-ARL3/miR-1305 axis and ultimately facilitating HCC progression (28).

METTL3 regulates m6A modifications of the oncogenes lncRNA GBAP1, LINC00958, Lnc-CTHCC, and MAPKAPK5_AS1 (MAAS) and subsequently promotes the progression of HCC (29–31). The expression of the lncRNA GBAP1 is significantly increased in HCC tissues (29). METTL3 induces the expression and stability of the lncRNA GBAP1 in HCC cells and promotes the migration, invasion, and proliferation of HCC cells through the miR-22-3p/BMPR1A/SMAD pathway (29). Heparin binding growth factor (HDGF) has been identified as an HCC oncogene that affects cellular lipid metabolism (30). METTL3 enhances the stability of LINC00958 through m6A modification and promotes lipogenesis through the miR-3619-5p/HDGF axis, ultimately contributing to HCC proliferation, migration and invasion (30). METTL3-mediated m6A modification in lnc-CTHCC is recognized by (IGF2BP1)/IGF2BP3, which maintains the stability of lnc-CTHCC and promotes HCC growth and metastasis through the lnc-CTHCC/hnRNPK/YAP1 axis (31). MAAS is an oncogene whose expression is upregulated in HCC cancer tissues, and its high expression is closely associated with a low likelihood of patient survival (32). Hepatitis B e antigen secreted by HCC cells upregulates MAAS expression in M2 macrophages by promoting METTL3-mediated m6A modification. MAAS is upregulated in HCC cells via M2 macrophage-derived exosomes and targets the MYC proto-oncogene to promote HCC cell proliferation (32).

Phosphatase and tensin homolog (PTEN) is a tumor suppressor that reduces carcinogenesis by inhibiting the PI3K/AKT pathway (33). HBV increases the m6A methylation of PTEN RNA through the regulation of METTL3, which leads to a decrease in its protein level and promotes HCC (33). Abnormal spindle-like microcephaly (ASPM) has been shown to be involved in tumor progression, and its high expression in HCC predicts a poor prognosis (34). METTL3-mediated m6A modification promotes the expression of ASPM, providing a new therapeutic strategy against HCC (34). Another study suggested that METTL3 may also be associated with lenvatinib resistance in HCC cells, driving cancer cell resistance through the METTL3-M6a/EGFR-pak2-erk5 axis; thus, METTL3 may be a potential therapeutic target for drug resistance (35).

The function of METTL3 is closely associated with its acetylation. When METTL3 undergoes substantial acetylation, its binding to metal-responsive transcription factor 1 (MTF1) mRNA, METTL14, and WTAP weakens, resulting in a decrease in m6A modification induced by the METTL3-METTL14-WTAP methyltransferase complex (36). In HCC, reduced m6A modification of MTF1 mediated by METTL3 acetylation leads to enhanced MTF1 expression, thereby promoting cell proliferation and tumor progression (36). Furthermore, mitogen stimulation leads to an increase in the small ubiquitin-related modifier (SUMO) ylation of METTL3, which is correlated with the upregulation of ubiquitin-conjugating enzyme 9 (UBC9) and is positively associated with the high metastatic potential of liver cancer (37). The UBC9/SUMOylated METTL3/Snail axis represents a novel pathway for SUMO involvement in HCC progression (37).

##### 3.1.1.2 METTL14

As a homolog of METTL3, METTL14 shares some similarities, but METTL14 is downregulated in HCC, is closely associated with tumor metastasis, and plays a regulatory role in the process of HCC tumor metastasis (**Figure 3**, **Table 1**) (39).

METTL14 can inhibit circSTX6 expression via m6A modification (38). Downregulation of METTL14 dysregulates the circSTX6/HNRNPD/ATF3 axis, accelerates HCC proliferation and tumorigenicity, and enhances tumor metastasis (38). Notably, the circSTX6-encoded protein circSTX6-144aa also independently promoted HCC progression and is expected to be a potential biomarker and therapeutic target for HCC (38).

Ubiquitin-specific peptidase 48 (USP48) is a member of the ubiquitin-specific protease family and has been identified as an inhibitor of HCC tumorigenesis by stabilizing sirtuin 6 (SIRT6) (39). The downregulation of METTL14 in HCC leads to decreased methylation levels of USP48 mRNA, resulting in reduced expression of USP48. This dysregulation consequently affects glycolysis through the USP48-SIRT6 axis and contributes to the deterioration of HCC (39). These findings indicate that specifically targeting hepatocyte USP48 or the USP48-SIRT6 axis may be a potential therapeutic strategy for future HCC treatment.

Hepatocyte nuclear factor 3γ (HNF3γ) is a hepatocyte nuclear factor that can inhibit HCC growth by transactivating organic anion transporting polypeptide 1B1 (OATP1B1) and 1B3 (OATP1B3) expression, which sensitizes HCC cells to sorafenib-induced growth inhibition and apoptosis (40). METTL14-mediated m6A modification plays an important role in maintaining high HNF3γ expression, and downregulation of METTL14 in HCC cells decreases HNF3γ expression and promotes HCC progression (40). Another study showed that high levels of METTL3/14 enhanced the expression of ATP citrate lyase (ACLY) and stearoyl-CoA desaturase 1 (SCD1) by regulating their mRNA stability, which accelerated fatty acid synthesis and lipogenesis, ultimately leading to lipid peroxidation or endoplasmic reticulum stress, resulting in HCC cell death and a decrease in HCC cell viability (42). Targeting METTL3/14 has proven to be a promising anticancer therapeutic strategy.

##### 3.1.1.3 Other “writers” and HCC

The M6A methylases WTAP and KIAA1429, also known as tumor-associated proteins, are significantly upregulated in HCC (43). WTAP can cause posttranscriptional repression of ETS proto-oncogene 1 (ETS1) through m6A modification and promote the proliferative capacity and tumor growth of HCC cells through the WTAP/ETS1-p21/p27 axis (43). The micropeptide encoded by lncRNA AC115619 inhibited the growth of HCC tumors by binding to WTAP and hindering the assembly of the m6A methyltransferase complex, resulting in a reduction in the overall methylation level (45). In addition, when researchers knocked out the WTAP gene in HCC, the m6A level of liver kinase B1 (LKB1) mRNA decreased, and its stability increased, which in turn promoted autophagy in HCC cells via the WTAP/LKB1/AMPK axis, suggesting that it is a promising target for HCC therapy (44). KIAA1429 was found to be significantly upregulated in HCC tissues. It has been demonstrated to promote the invasion, migration, and EMT of sorafenib-resistant HCC cells (46). Additionally, through its mediation of m6A methylation, KIAA1429 was observed to decrease the expression of the E-Ca protein while increasing the expression of the slug and snail proteins (46).

#### 3.1.2 The “erasers” and HCC

##### 3.1.2.1 ALKBH5

ALKBH5 is a demethylating enzyme with extremely complex regulatory mechanisms *in vivo* (67). The expression of ALKBH5 was reported to be downregulated in HCC, causing elevated m6A levels and increased stability of Ly6/Plaur domain-containing 1 (LYPD1), leading to dysregulation of the ALKBH5/LYPD1 axis and promoting the progression of HCC (48). DEAD-box helicase 24 (DDX24) is a tumor-associated gene that is often used as a marker to assess tumor risk and treatment efficacy (49). Downregulation of ALKBH5 promotes the expression of LINC02551, which acts as a molecular junction to block the binding of DDX24 to the E3 ligase tripartite motif 27, thereby reducing the ubiquitination and subsequent degradation of DDX24 (49). Moreover, stabilized DDX24 promotes EMT in HCC (49). However, some researchers have reported conflicting results. In contrast to the aforementioned study, they proposed that ALKBH5 is actually upregulated in HCC. They suggested that this upregulation occurs through the activation of the ERK/JNK pathway and the regulation of interleukin-8 (IL-8) expression via the ALKBH5/MAP3K8 axis. Ultimately, this promotes HCC development, metastasis, and macrophage recruitment (50). According to reports, there is a strong positive correlation between HBx and ALKBH5 in HBV-HCC tissues (51). It has been discovered that HBV increases the expression of ALKBH5 through the HBx/wdr5/h3k4me3 pathway, while ALKBH5, in turn, enhances the stability of HBx mRNA by reducing m6A modification (51). Additionally, studies have indicated that the depletion of ALKBH5 significantly suppresses the proliferation and migration of HBV-induced tumor cells both *in vitro* and *in vivo* (51).

##### 3.1.2.2 Other “erasers” and HCC

FTO plays key roles in the regulation of adiposity, lipogenesis, and body weight (97). The expression level of FTO is elevated in HCC, which is promoted by the lncRNA FTO-IT1 recruiting interleukin enhancer binding factor 2 (ILF2) and 3 (ILF3) (52). FTO overexpression increases the mRNA expression of the glycolysis-associated genes glucose transporter type 1 (GLUT1), pyruvate kinase M2 (PKM2), and c-Myc by decreasing the expression of m6A modifications, which subsequently promotes HCC progression in a glycolysis-dependent manner (52). Sirtuin 1 (SIRT1) is a known deacetylase silencing information regulator that destabilizes FTO, and its presence is positively correlated with malignancy and metastasis (53). A reduction in FTO by SIRT1 can increase the m6A modification level of guanine nucleotide-binding protein G (o) subunit α (GNAO1) and cause downregulation of its mRNA expression (53). Deletion of GNAO1 significantly enhanced HCC proliferation and invasion *in vitro* (53).

RALY RNA Binding Protein Like (RALYL) is a recently discovered demethylase that can increase the stability of TGF-β2 mRNA by reducing its m6A modification (54). RALYL promotes the tumorigenicity, self-renewal, chemoresistance, and metastasis of HCC through the TGF-β2/PI3K/AKT and STAT3 pathways (54). Zinc finger CCCH-type containing 13 (ZC3H13) is a methyltransferase whose expression is downregulated in HCC (55). The miR-362-3p/miR-425-5p-ZC3H13 axis leads to downregulation of ZC3H13 and thus a reduction in m6A modifications, which correlates with poor prognosis and poor outcome in HCC patients (55).

#### 3.1.3 The “readers” and HCC

##### 3.1.3.1 IGF2BP1

IGF2BP1, a common m6A methylation recognition protein that recognizes a wide range of m6A-modified RNAs, is highly expressed in HCC (59). The expression of autophagy related 16 like 1 (ATG16L1), a member of the autophagy family, is increased in both HCC cells and tissues. This finding suggested that autophagy may play a role in the progression of HCC (56). IGF2BP1 can recognize circMDK via m6A sites, activate the PI3K/AKT/mTOR signaling pathway through the miR-346/874-3p-ATG16L1 axis, and ultimately promote the proliferation, migration and invasion of hepatoma cells (56). IGF2BP1 recognizes the m6A modification of circMAP3K4 and promotes its translation, thereby preventing cisplatin-induced apoptosis in HCC cells by increasing the interaction of circMAP3K4-455 aa with apoptosis inducing factor (AIF) (57). High levels of circMAP3K4 serve as an independent prognostic factor for poor overall survival and disease-free survival in HCC patients (57). Furthermore, IGF2BP1 is capable of recognizing the m6A-modified lncRNA MIR4435-2HG and enhancing its expression in HCC (58). Moreover, overexpression of MIR4435-2HG significantly reduces cell sensitivity to lenvatinib, enhances the stem cell properties of HCC cells, and promotes tumorigenesis *in vitro* and *in vivo* (58). Additionally, IGF2BP1 enhances alpha-1,6-mannosylglycoprotein 6-beta-N-acetylglucosaminyltransferase V (MGAT5) mRNA stability by increasing m6A modification, which contributes to the promotion of the HCC stem cell phenotype, self-renewal, chemotherapy resistance, and tumorigenesis in mice (59). In conclusion, IGF2BP1 is highly expressed in HCC and enhances the translational stability of corresponding HCC-associated RNAs by recognizing the m6A site. This finding suggests that it may be an important target for anticancer therapy.

##### 3.1.3.2 YTHDF family

The YTHDF family is classified as m6A recognition proteins and consists of YTHDF1, YTHDF2, and YTHDF3. These proteins have been linked to the progression of HCC (60–63). Under hypoxic conditions, hypoxia-inducible factor-1α (HIF-1α) promotes the expression of YTHDF1 by directly binding to its promoter region. This mechanism facilitates hypoxia-induced autophagy in HCC and autophagy-related malignancies through the HIF-1α/YTHDF1/ATG2A/ATG14 axis (60). Additionally, YTHDF2 regulates the m6A methylation of octamer-binding transcription factor 4 (OCT4) mRNA, thereby promoting a cancer stem cell (CSC) liver phenotype and tumor metastasis (61). Targeting YTHDF2 to eliminate CSCs is a key focus in the development of novel anticancer therapies (61). The m6A modification mediated by YTHDF3 has been shown to promote HCC migration, invasion, and EMT processes, underscoring its potential as a promising therapeutic target for HCC (62, 63). Additionally, Circ_KIAA1429 has been identified as an oncogene that targets zinc finger E-box binding homeobox 1 (Zeb1) downstream of HCC. Its high expression level has been associated with a lower overall survival rate in HCC patients (62). Through M6A-dependent mechanisms, YTHDF3 enhances Zeb1 mRNA stability and promotes HCC progression through the TYHDF3/Zeb1/KIAA1429 axis (62). Furthermore, YTHDF3 plays a role in enhancing the translation and stability of m6A-modified epidermal growth factor receptor (EGFR) mRNA. This stimulation further drives HCC progression through the YTHDF3/m6A EGFR/STAT 3 and EMT pathways (63).

##### 3.1.3.3 LRPPRC

Programmed cell death ligand 1 (PD-L1) is a transmembrane protein expressed on the surface of activated T cells, B cells, and natural killer cells, and acts as a ligand for programmed cell death protein 1 (PD-1) and is commonly found on the surface of tumor cells (98). The interaction between these two proteins can effectively suppress the activation and proliferation of T cells, diminish the cytotoxic capabilities of T-cells towards tumor cells, and promote immune evasion and tumor cell growth (65). PD-L1 plays a crucial role as a coinhibitory immune checkpoint, and the PD1/PD-L1 signaling pathway functions to dampen the cytotoxic T-cell-mediated killing effect within the tumor microenvironment, thereby aiding in immune evasion by tumors (65). Research has indicated that LRPPRC is upregulated in HCC and enhances PD-L1 expression through a m6A-mediated mechanism. This process leads to increased stability of PD-L1 mRNA in cancer cells, ultimately promoting tumor growth and facilitating immune evasion and invasion by tumor cells (65).

### 3.2 m5C in HCC

NSUNs are a class of m5C methyltransferases in which NSUN2 and NSUN4 are upregulated and NOP2 is downregulated in HCC tissues and cells (77, 78, 99). The expression of NSUN4 varies across different survival rates and grade distributions, indicating its potential as an independent prognostic factor for HCC (99).

NSUN2-mediated gene mRNA and lncRNA H19 m5C modifications regulate the progression of HCC (77, 78). In HCC tissues, the m5C modification level of mRNA is significantly greater than that in normal tissue, and growth factor receptor-bound protein 2 (GRB2), ring finger protein 115 (RNF115) and apoptosis antagonizing transcription factor (AATF) are the top NSUN2-related m5C hypermethylated genes (78). GRB2 is a growth factor receptor-binding protein that is expressed at significantly higher levels in HCC tissues than in normal tissues and is considered a potential therapeutic target for HCC (78). NSUN2 interacts with lin-28B, a protein capable of recognizing m5c, to facilitate the m5c modification of GRB2 and enhance the stability of GRB2 mRNA. This process ultimately contributes to promoting resistance in HCC cells to sorafenib by activating the Ras and p-Erk pathways (78). H19, an important tumor-related lncRNA, is targeted by NSUN2 in HCC (77). The NSUN2-mediated lncRNA H19 m5C modification enhances its stability and increases its ability to bind specifically to Ras-GTPase activating SH3 domain-binding protein 1 (G3BP1) to promote the proliferation, migration and invasion of HCC cells (77).

NOP2, also known as NSUN1, is a member of the NSUN family whose expression is downregulated in HCC (79). NOP2 overexpression enhances the expression of the antioncogene xeroderma pigmentosum D (XPD) via m5C methylation of XPD, thereby inhibiting the proliferation, migration and invasion of HCC cells (79). These findings suggest that XPD may be a potential target for HCC treatment.

ALYREF is a protein that recognizes m5C and is upregulated in HCC (100). The EGFR signaling pathway plays a crucial role in various cellular processes, and abnormal activation of EGFR is observed in a wide range of tumors (100). ALYREF stabilizes EGFR by binding to the m5C site on EGFR mRNA, thereby activating STAT3 signaling and promoting HCC progression (80).

### 3.3 m1A in HCC

m1A modification is involved in the progression and treatment of HCC (**Table 2**). In HCC tissues, the m1A modification levels of RNA are aberrantly elevated (81). The expression levels of m1A methyltransferases, such as TRMT6 and TRMT61A, are significantly elevated in HCC tissues and are negatively correlated with patient prognosis (81). The PPARδ protein, also known as peroxisome proliferator-activated receptor, is associated with cholesterol synthesis (81). TRMT6/TRMT61A enhances m1A methylation in tRNA, leading to an increase in PPARδ translation. This subsequently promotes cholesterol synthesis and activates Hedgehog signaling, ultimately driving self-renewal and tumorigenesis of liver CSCs (81).

In addition, the m1A demethylase ALKBH3 is significantly upregulated in HCC tissues compared to nontumor tissues. Furthermore, high expression of ALKBH3 has been associated with poor prognosis (43). The overexpression of ALKBH3 has been found to stimulate the proliferation and tumorigenesis of HCC tumor cells, indicating a functional role for m1A modification in promoting the cell cycle (82).

For binding proteins that specifically recognize the m1A site, members of the YTH domain family, such as YTHDF1, YTHDF2, and YTHDF3, have been reported to be upregulated in HCC tissues compared to normal tissues (83). Additionally, the expression levels of YTHDF1, YTHDF2, and YTHDF3 are positively correlated with immune cell infiltration in HCC tissues. This finding indicates a functional role for m1A regulators in regulating immune cell infiltration (83).

In addition, there have been reports suggesting that patients with smaller tumors and good liver function may benefit from a combined regimen of mitoxantrone, 5-fluorouracil, and cisplatin (90). The clinical use of doxorubicin in patients with elevated levels of m1A modification is more scientifically supported. These findings contribute to mitigating the risk of doxorubicin-induced cardiotoxicity in patients and reducing unnecessary overtreatment (90).

### 3.4 m7G in HCC

Methylation of m7G has been found to be associated with the progression of HCC and resistance to drugs (**Table 2**). Both METTL1 and WDR4, which are essential components of the m7G methyltransferase complex, have been reported to be upregulated in HCC tissues and cells (84, 86). In HCC cells, MYC activates the transcription of WDR4, which subsequently enhances the stability and translation of cyclin B1 (CCNB1) mRNA by facilitating binding of eukaryotic initiation factor 2A (EIF2A) to CCNB1 mRNA. This process ultimately promotes proliferation, metastasis, and resistance to sorafenib (84, 86). Furthermore, METTL1-mediated modification of tRNA m7G contributes to hepatocarcinogenesis through translational control of target mRNAs (86).

Long-term drug resistance is a significant challenge in the treatment of HCC. Studies have indicated that METTL1 and WDR4 are upregulated in lenvatinib-resistant cells (87). METTL1/WDR4-mediated tRNA m7G modification enhances the translation of genes within the EGFR pathway, ultimately leading to lenvatinib resistance in HCC cells. This suggests a potential strategy for overcoming drug resistance (87). In glioblastoma, tripartite motif containing 28 (TRIM28) has been identified as a specific marker of stem-like cells, contributing to their invasion (85). Additionally, WDR4 amplifies TRIM28 expression, subsequently affecting the expression of target genes and promoting cell-acquired stemness as well as lenvatinib resistance (85).

Insufficient radiofrequency ablation (IRFA) is a major contributing factor to the high recurrence rate of HCC treatment (88). Studies have shown an association between m7G-tRNA modification and HCC recurrence following IRFA treatment (88). Specifically, after IRFA therapy, there was a significant increase in the level of m7G tRNA modification and its associated methyltransferase complex component METTL1/WDR4. This increase facilitated the translation of SLUG/SNAIL during sublethal heat stress in a manner dependent on codon frequency, ultimately leading to elevated HCC recurrence and metastasis (88).

In summary, RNA modifications, such as m6A, m5C, m1A, and m7G, through a series of modification regulatory proteins regulate the expression of HCC-associated genes and are involved in the progression of HCC via different signal axes. It is important to emphasize that the alterations in the expression of RNA modification regulatory proteins with similar functions in HCC are somewhat inconsistent. Methyltransferases, such as METTL3 and METTL14, as well as USUN2 and NOP2, exhibit opposite expression changes in HCC. Their opposite expression changes play a similar role in the progression of HCC by regulating the expression of different target genes and subsequent signaling pathways through RNA modification. There is no consensus on the expression changes of some RNA modification regulatory proteins, such as ALKBH5 and FTO, in HCC. This suggests that the expression of RNA modification regulatory proteins in HCC may be dynamic and have different mechanisms of action in various stages and types of the HCC. Therefore, more in-depth and detailed classification research is needed to clarify this.

At present, research on the role of RNA modification in HCC mainly focuses on m6A, m1A, m5C, m7G, etc. Their combined interactions form an intricate RNA modification network that significantly influences the physiological and pathological processes of HCC cells. However, whether these RNA modifications interact with each other in HCC has not been studied yet. The interactions between different types of RNA modifications are complex and diverse, and they may affect each other through synergistic effects, competitive relationships, cascade effects (101). Both m6A and inosine occur at the N6 position of the adenosine ring, but they do not compete for the same adenosine (102). m5C and m6A work together to regulate the export of mRNA to the cytoplasm through interactions with ALYREF and ALKBH5 (101). The m6A and m1A modifications on mRNA are recognized by the same reader proteins YTHDF1-3. In addition, m7G plays a role in facilitating the N6, 2-O-dimethyladenosine modification within the cap structure of the extended RNA polymerase II transcript (103). Despite the specific interplay between RNA modifications not being fully elucidated, further research is anticipated to unveil more about the mechanism and function of these modifications, offering new insights and approaches for the treatment and diagnosis of diseases.

## 4 Potential applications of RNA modifications in HCC

### 4.1 Potential for use as diagnosis and prognosis biomarkers

Proteins and genes associated with RNA modifications have the potential to serve as biomarkers for the diagnosis and prognostic evaluation of HCC (**Table 3**). This capability has the potential to improve diagnostic accuracy and ultimately enhance the survival rate of patients.

**Table 3:** Potential for use as diagnosis and prognosis biomarkers of HCC.

Biomarker	Expression	Diagnosis (AUC)	Prognosis (HR)	Treatment decision utility	REF
KIAA1429	Up	HCC diagnosis (TCGA: 0.85)	Poor prognosis (>1)	Promote sorafenib resistance	(46, 47)
WTAP	Up	–	Poor prognosis (>3)	–	(43)
METTL14	Down	–	Increased likelihood and severity (>2)	Weaken sorafenib response	(39–41)
METTL3	Up	–	Increased likelihood and severity (>1)	Promote lenvatinib resistance	(35, 37)
ALKBH5	Down	HCC diagnosis (-)	Poor prognosis (>2)	–	(48)
FTO	Up	HCC diagnosis (-)	Poor survival (>1)	–	(52, 104)
	Down	–	Poor survival (>1)	–	(105)
RALYL	Up	–	Poor survival (>4)	Enhance cisplatin and 5-fluorouracil resistance	(54)
ZC3H13	Down	–	Poor survival(>1)	–	(55)
IGF2BP1	Up	–	Poor survival (>1)	Promote lenvatinib resistance	
YTHDF1	Up	–	Poor prognosis (>1)	–	(60)
YTHDF2	Up	–	Poor prognosis (>1)	–	(61)
YTHDF3	Up	–	Poor prognosis (>5)	–	(62, 64)
LRPPRC	Up	–	Poor prognosis (>1)	–	(65)
NSUN2	Up	–	Increased likelihood and severity (>1)	–	(78)
ALYREF	Up	HCC diagnosis (-)	Poor prognosis (>2)	–	(80, 106)
METTL1	Up	–	Increased likelihood and severity (>1)	Promote lenvatinib resistance	(87)
WDR4	Up	–	Increased likelihood and severity (>1)	Promote sorafenib resistance	(84)

HCC, hepatocellular carcinoma; REF, references; AUC, area under receiver operating characteristic curve; HR; hazard ratio of overall survival; TCGA, the cancer genome atlas.

#### 4.1.1 Role of m6A regulators in HCC diagnosis, prognosis and drug resistance

The expression levels of KIAA1429, ALKBH5 and FTO were reported to have the diagnostic potential in HCC (**Table 3**). A study found that KIAA1429 expression had up to 0.85 of the area under the curve (AUC) in the receiver operating characteristic curve (ROC) analysis, which indicated that it has a relatively high diagnostic value in HCC (47). Although studies have suggested the potential diagnostic potential of ALKBH5 and FTO, their potential and value as diagnostic markers have been reduced due to inconsistent expression in different studies (52, 104, 105) and a lack of ROC analysis data for diagnostic markers (48).

For the upregulated m6A methyltransferases in HCC cells, the higher expression levels of KIAA1429, WTAP, and METTL3 are associated with poorer overall survival outcomes and indicate a poor prognosis and increased likelihood and severity of patients (35, 37, 43, 46). The micropeptide AC115619-22aa encoded by lncRNA AC115619, as an inhibitor of WTAP, is also a potential prognostic indicator for HCC (36). Additionally, the elevated expression of ASPM facilitated by METTL3 via m6A modification is also strongly associated with a poor prognosis of HCC (34). The methyltransferase METTL14 is downregulated in HCC, and lower expression levels of METTL14 are associated with increased likelihood and severity of HCC patients who underwent a poorer overall survival (41). Besides, there are many pathways downstream of METTL14 that increase the likelihood and severity of HCC (107). Reduced HNF3γ expression is associated with the malignant features of HCC and is correlated with poor patient survival (40). CircSTX6 and its encoded proteins are expected to have the potential to serve as biomarkers for the diagnosis, prognosis, and treatment of HCC (38).

Low expressions of demethylases ALKBH5 and ZC3H13 are associated with poor prognosis in HCC patients (48, 50, 55). Additionally, the overexpression of ALKBH5 has been found to cause a decrease in the level of the lincRNA LINC02551, which has been utilized as a prognostic biomarker for HCC (49). Furthermore, the overexpression of the demethylase RALYL has been associated with a poorer prognosis, lower levels of differentiation, and an increased likelihood of metastasis in clinical HCC patients (54). The expression of FTO varies in different studies, with one study showing high expression and another showing low expression, both of which were linked to poorer survival in HCC patients (52, 104, 105). These conflicting findings indicate the multifaceted and functionally complex nature of FTO in HCC, highlighting the need for further research to elucidate its molecular mechanism. The deacetylase SIRT1 reduces the expression of the m6A demethylase FTO, thereby increasing the m6A levels of its downstream target GNAO1 and downregulating its mRNA expression during HCC tumorigenesis (53). The discovery of potential diagnostic biomarkers such as SIRT1, FTO, and GNAO1 offers a promising direction for future research. This area holds significant promise for further investigation and exploration (53).

The significant overexpression of the m6A reading proteins IGF2BP1, YTHDF1, YTHDF2, YTHDF3, and LRPPRC in HCC tissues has been associated with a poorer prognosis for patients (**Table 3**). IGF2BP1 can promote the progression of HCC through the m6A-mediated upregulation of circMDK and circMAP3K4, which are associated with poor survival in HCC patients and serve as potential tumor biomarkers (56, 57). Furthermore, the results of multivariate Cox regression analysis indicated that the expression of YTHDF1 served as an independent prognostic factor for patients with HCC (60). In addition, scientists have shown a negative correlation between YTHDF2 expression and patient survival (61). Therefore, YTHDF2 plays a significant role in the oncogenesis of HCC and can serve as a valuable biomarker for HCC patients. Additionally, the expression level of YTHDF3 in HCC tissues was found to be significantly greater than that in adjacent liver tissues (62). YTHDF3 plays a crucial role in regulating the progression of HCC, providing a promising new target for HCC treatment (63). In conclusion, the YTHDF family is closely associated with the prognosis of HCC patients and may represent a potential target for the future treatment of HCC. Additionally, LRPPRC is frequently upregulated in HCC tumors, which is linked to advanced disease stages and poor prognosis (65).

Overall, about thirteen m6A regulators are reported to be closely related to HCC prognosis and have a potential for use as prognosis biomarkers, including four methyltransferases (KIAA1429, WTAP, METTL14 and METTL3), four demethylases (ALKBH5, FTO, RALYL and ZC3H13), and five reading proteins (IGF2BP1, YTHDF1-3 and LRPPRC (**Table 3**). Among them, the high expression of WTAP, RALYL, YTHDF3, etc. showed a higher hazard ratio of overall survival (>3) in survival analysis, indicating a more significant correlation and impact on poor survival rates, suggesting that these markers have relatively higher prognostic value (**Table 3**). It should be noted that the expression changes of ALKBH5, and FTO in HCC vary in different studies, and these contradictory results need further research and clarification. In addition, the differences in the patient source, quantity, and calculation method of the survival curve analysis of these regulatory proteins indicate strong heterogeneity. Therefore, the confirmation of the prognostic value of each factor still requires multi-center large-scale sample validation. Confirming the prognostic performance of these potential markers through further ROC curve analysis of large sample sizes is an important research direction for the future. In addition, most previous studies have analyzed these factors as independent prognostic factors for patients with HCC, attempting to conduct a joint analysis of these factors for prognosis, which may provide a more comprehensive display of the relationship between m6A regulators and HCC prognosis.

In addition to patient survival rates, some m6A regulators also exhibit a correlation with targeted anti-cancer drugs or chemotherapy drugs, promoting cancer cell drug resistance (**Table 3**). High expression of KIAA1429 and low expression of METTL14 can promote sorafenib resistance in HCC cells. KIAA1429 is involved in promoting invasion, migration, and EMT in sorafenib-resistant HCC by mediating m6A methylation (21). THNF3γ reduction caused by METTL14 knockdown upregulates the expression of the sorafenib influx transporters OATP1B1 and OATP1B3, thereby rendering sorafenib resistance in HCC, and enforced HNF3γ expression enhances the cellular response to sorafenib in HCC (40). Similarly, high expression of METTL3 and IGF2BP1 can promote lenvatinib resistance in HCC cells, while high expression of RALYL enhances cisplatin and 5-fluorouracil resistance. Targeting METTL3 with the specific inhibitor STM2457 improved sensitivity to lenvatinib in both *in vitro* and *in vivo* studies (35). This discovery suggested that METTL3 might serve as a potential therapeutic target for overcoming lenvatinib resistance in HCC. There have been reports suggesting that patients with smaller tumors and good liver function may benefit from a combined regimen of mitoxantrone, 5-fluorouracil, and cisplatin (108). Inhibiting RALYL could potentially improve the effectiveness of a combination therapy involving mitoxantrone, 5-fluorouracil, and cisplatin, providing new strategies for treating HCC. These results not only provide a theoretical basis for personalized medication for patients but also offer new directions for increasing drug sensitivity research. The combination of specific inhibitors or downstream target genes that modify related proteins with chemotherapy drugs may provide new strategies for the treatment of HCC.

#### 4.1.2 Role of m5C regulators in HCC diagnosis, prognosis and drug resistance

Regulators of RNA m5C modification and their target HCC-related oncogenes are potential diagnostic and prognostic biomarkers for HCC (**Table 3**). A significant increase in the expression of lncRNA H19 was reported to be associated with the development of various types of tumors (77). The RNA m5C methyltransferase NSUN2 regulates the stability of lncRNA H19 through m5C modification and may serve as a potential new target and biomarker for the treatment of HCC (77). In addition, NSUN2 has been found to impact the sensitivity of HCC cells to sorafenib by regulating the activity of the Ras pathway (78). The sensitivity of NSUN2 knockout cell lines to sorafenib was significantly greater than that of control cells (78). ALYREF, functioning as a “reader” for the m5C site in RNA, is a protein that shuttles between the nucleus and cytoplasm, and plays a critical role in facilitating the transportation of RNA from the nucleus to the cytoplasm. The upregulation of ALYREF has been associated with poor prognosis in HCC patients, indicating its potential as a valuable target for diagnosing and predicting prognosis in HCC patients (80, 100). The higher HR value of ALYREF than NSUN2 suggests a priority prognostic value of ALYREF. However, due to the lack of necessary ROC analysis, further analysis of large sample sizes is needed to confirm the prognostic or diagnostic values of NSUN2 and ALYREF.

#### 4.1.3 Role of m7G regulators in HCC prognosis and drug resistance

The upregulation of the m7G methyltransferases METTL1 and WDR4 has been reported to be correlated with advanced tumor stage and unfavorable patient survival outcomes in the literature (86). Investigators have shown that METTL1-mediated tRNA m7G modification plays a critical role in lenvatinib resistance in HCC cells by enhancing the translation of EGFR pathway genes (87). WDR4 overexpression significantly increased the half-maximal inhibitory concentration value of sorafenib in HCC cells (77). Some researchers propose that both METTL1 and WDR4 have the potential to serve as biomarkers for predicting the efficacy of lenvatinib and sorafenib (87). The potential application of m7G regulators in HCC is still in the early stages of research. Further exploration of new m7G regulators involved in the occurrence and progression of HCC may provide new targets for the prognosis and treatment of HCC.

#### 4.1.4 Potential applications of non-coding RNAs regulated by RNA modification

Non-coding RNAs, as important target sequences regulated by RNA modifications, play a crucial role in the pathogenesis of HCC (**Figure 3**). Upregulated circRNAs dependent on M6A modification, including circ-CCT3, circSTX6, circ-ARL3, circMDK, circMAP3K4, and circ_KIAA1429, are involved in the proliferation, invasion, migration, and apoptosis of HCC cells. Upregulated lncRNAs dependent on m6A modification, such as GBAP1, lnc-CTHCC, LINC00958, MIR4435-2HG, etc., play similar functional roles in the progression of HCC. Certain non-coding RNAs regulated by RNA modification showed significant application potential in the diagnosis, prognosis, and treatment of HCC. The elevated expression of circSTX6 has an AUC of up to 0.8565 in the diagnostic ROC analysis, suggesting a considerable diagnostic significance in HCC (38). The high expression of LINC00958, lnc-CTHCC showed a higher hazard ratio of overall survival (>2) in survival analysis, indicating a more significant correlation and impact on poor survival rates, suggesting that these markers have relatively higher prognostic value (30, 31). Overexpression of MIR4435-2HG significantly reduced cell susceptibility to lenvatinib, suggesting its role in cell resistance to lenvatinib (58). Nevertheless, most research has focused solely on investigating the impact of partial non-coding RNA in m6A modification, leaving a gap in understanding the connection between miRNA and RNA modification, along with the correlation between m5C, m1A, m7G, and RNA modification. This presents a promising avenue for further investigation.

### 4.2 Potential agents targeting RNA modification

#### 4.2.1 Potential agents targeting m6A

**Figure 4:**
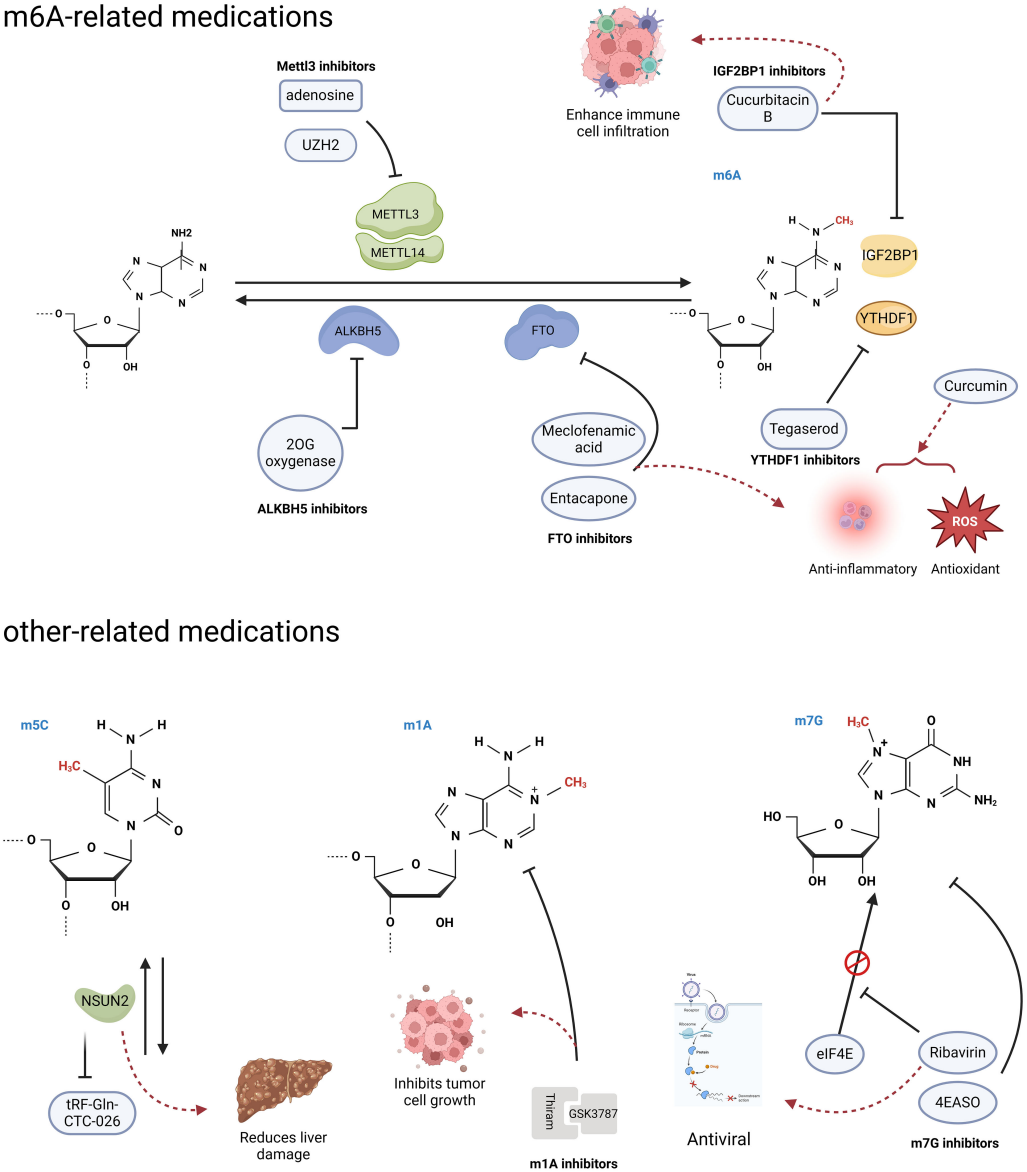
RNA modification-related agents. Research has found that some agents act on RNA modified regulators, and can trigger a series of downstream effects. (Created with BioRender.com).

As the link between RNA modifications and cancer continues to be discovered, the demand for inhibitors of the associated proteins is increasing. The role of the m6A methyltransferase METTL3 in various diseases is continuously expanding, and there is also a growing focus on the development of METTL3 inhibitors. This has garnered increasing attention in academic research and scholarly discourse (**Figure 4**). Adenosine was the first reported METTL3 inhibitor that acts in a SAM-competitive manner to reduce the level of m6A modification (109). UZH2 is a small molecule inhibitor that selectively targets METTL3 and reduces the m6A levels of polyadenylated RNA in the MOLM-13 (acute myeloid leukemia) and PC-3 (prostate cancer) cell lines and has great potential as a therapeutic agent for cancer (110, 111). Curcumin, a polyphenolic pigment derived from turmeric, is yellow in color and has been reported to exhibit anti-inflammatory, anticancer, antioxidant, and antibacterial activities (112). Curcumin can increase the level of m6A modification in piglet liver by affecting the mRNA expression of METTL3, METTL14, ALKBH5, FTO, and YTHDF2 and subsequently attenuate polysaccharide-induced disorders of hepatic lipid metabolism (113).

FTO, a m6A demethylase, has been reported to play important roles in various diseases, including HCC and diabetes mellitus, and is a potential therapeutic for related diseases (114). Entacapone and meclofenamic acid (MA) are reported to be inhibitors of the m6A demethylase FTO (**Figure 4**) (114–116). As a chemical inhibitor, entacapone inhibits FTO-mediated metabolic regulation through forkhead box O1 (FOXO1) (114). MA, a nonsteroidal anti-inflammatory drug, has been identified as a highly selective FTO inhibitor that competitively binds to FTO, thereby enhancing overall m6A modification levels (97). In the case of inhibitors targeting another m6A demethylase, ALKBH5, only a small number of nonselective compounds have been discovered (**Figure 4**). The majority of these compounds are 2OG oxygenase inhibitors, with only a limited few characterized as ALKBH5 inhibitors. Currently, there is a lack of significant selective inhibitors for ALKBH5 (117).

In addition, Hong et al. conducted a structure-based drug screening and identified tegaserod as a potential inhibitor of YTHDF1 (**Figure 4**) (118). Tegaserod functions by blocking the binding of YTHDF1 to m6A-modified mRNA, thereby inhibiting the YTHDF1-mediated translation of Cyclin E2. This suggests that tegaserod may serve as a promising antitumor agent (119). Modulation of the tumor immune microenvironment has emerged as a novel approach for cancer immunotherapy in HCC, with m6A modification being an ideal target due to its role in the immune response (120). The expression of IGF2BP1, which recognizes m6A modifications, is significantly upregulated in HCC and is associated with immune cell infiltration (121). Cucurbitacin B has been demonstrated to inhibit the binding of IGF2BP1 to m6A-modified mRNA and increase immune cell infiltration, suggesting that it is a potential anti-HCC agent (**Figure 4**) (121).

#### 4.2.2 Potential agents targeting m5C

The m5C writing protein is a promising target for the treatment of HCC. The upregulation of the m5C methyltransferase NSUN2 in HCC has been observed (77). Knocking down NSUN2 leads to reduced levels of m5C modification, and some unmodified tRNAs undergo complex changes to become tfRNAs (tRF-Gln-CTC-026), which effectively alleviate liver injury by inhibiting global protein synthesis (GPS) (122). Efforts to deplete NSUN2 and provide protective tfRNAs are potential treatments for liver injury and have significant implications for reducing the risk of HCC occurrence.

#### 4.2.3 Potential agents targeting m1A

Thiram, a potent inhibitor of the m1A methyltransferase complex TRMT6/TRMT61A, has been shown to significantly inhibit the self-renewal of hepatic CSCs and hepatic tumor growth (**Figure 4**) (81). Furthermore, when combined with the PPARS antagonist GSK3787, thiram synergistically inhibits the development of HCC and the growth of tumors that are hypermethylated with m1A (81).

#### 4.2.4 Potential agents targeting m7G

Ribavirin is a widely used antiviral medication that has recently been shown to possess antitumor effects as a m7G cap analog that inhibits cell proliferation (**Figure 4**) (123). Additionally, the eIF4E antisense oligonucleotide drug (4EASO) is a well-established medication that competitively inhibits eIF4E binding to the m7G cap (124). These medications have shown promising results in halting cancer progression and improving prognosis, offering hopeful prospects for the development of additional m7G-targeted drugs.

## 5 Challenges and opportunities for RNA modification

Currently, research on the role of RNA modification in HCC mainly focuses on m6A, m1A, m5C, and m7G. The expression of RNA modification regulators, including “writer”, “eraser” and “reader” proteins, changed significantly in HCC, and are involved in the proliferation, autophagy, innate immunity, invasion, metastasis, immune cell infiltration, and drug resistance of HCC via different signal axes. The varying expression of RNA modification regulatory proteins with similar functions in HCC, along with the lack of consensus on the expression changes of some RNA modification regulatory proteins, indicates that the expression of these proteins in HCC may be dynamic and have different mechanisms of action in various stages and types of the disease. Consequently, further comprehensive and detailed classification research is necessary to elucidate this matter. Additionally, additional investigation is required to ascertain the relationships between these various forms of RNA modifications. In addition to the aforementioned modifications, there are still multiple types of RNA modifications involved in regulating post-transcriptional gene expression, including 5-hydroxymethylcytosine, pseudouridine, and 2’-O-methylation. Exploring whether these recently discovered RNA modifications are also involved in the occurrence and development of HCC may be a new research hotspot in the future. As high-throughput sequencing technology and novel biomarkers continue to advance, we will be able to delve deeper into the intricate regulatory network of RNA modifications in HCC, ultimately providing more precise and effective approaches for clinical diagnosis and treatment.

Studying RNA modifications in HCC will contribute to an in-depth understanding of the pathogenesis of HCC, the search for new diagnostic and prognostic markers, the development of new therapeutic strategies, and the assessment of treatment efficacy and the monitoring of recurrence. These findings provide a new perspective for the diagnosis and treatment of HCC by targeting specific RNA-modifying enzymes, recognition proteins, or related RNAs. More than ten RNA-modifying regulators showed the potential for use for the diagnosis, prognosis, and treatment decision utility biomarkers of HCC. KIAA1429 has a relatively high diagnostic value in HCC. For other potential diagnostic markers, further research is needed because of inconsistent expression in different studies and a lack of ROC analysis data for diagnostic markers. WTAP, RALYL, and YTHDF3 exhibit significantly higher prognostic significance in HCC. However, the validation of their prognostic value necessitates extensive multi-center sample validation and ROC curve analysis in the future. On the other hand, prior research has focused on examining these factors separately as prognostic indicators for HCC patients. Conducting a combined analysis of these factors could offer a more comprehensive understanding of the association between RNA-modifying regulators and HCC prognosis. Some RNA-modifying regulators also exhibit an association with targeted anti-cancer drugs or chemotherapy drugs, which provide a theoretical basis for personalized medication for patients. The concurrent administration of drugs and inhibitors of regulatory proteins based on the relationship between regulatory proteins and drug resistance may enhance the sensitivity of drugs and offer novel approaches for managing HCC.

A growing number of RNA modifier inhibitors are being developed, but the lack of preclinical experiments and clinical studies targeting RNA modification in HCC poses a significant obstacle, and further research is needed to evaluate their application value in HCC treatment. Studying RNA modifications in liver cancer necessitates careful consideration of the potential and challenges associated with its translational application. Although RNA modification may present a novel target for HCC treatment, the lack of specific drugs targeting RNA modification poses a significant obstacle. Many RNA modifications occur at low levels, making it difficult to selectively target and manipulate modified RNA molecules without affecting unmodified ones. Additionally, our understanding of the structural domains of certain RNA modifiers, such as those in the NSUN and TRMT families, remains limited. This lack of knowledge makes it even more challenging to design inhibitors that effectively target these proteins. Therefore, further investigation into the mechanism of action of RNA modification is imperative to identify effective strategies for regulating this process and to evaluate its safety and efficacy. The development of effective tools and techniques for targeting and manipulating specific RNA modifications is an ongoing challenge. Overcoming these obstacles will pave the way for the development of targeted therapies and interventions that can harness the potential of RNA modifications in various biological and disease settings.

## 6 Conclusion

RNA modifications play a crucial role in HCC progression. Specific RNA modification pathways, such as m6A, m5C, m1A, and m7G, are erroneously regulated by a series of modification regulatory proteins, thereby regulating the expression of HCC-associated genes, and are involved in the proliferation, autophagy, innate immunity, invasion, metastasis, immune cell infiltration, and drug resistance of HCC. These functional roles and molecular mechanism advancements of RNA modification offer new perspectives on the pathogenesis, as well as potential new diagnostic and prognostic markers and therapeutic strategies of HCC. At present, over ten RNA-modifying regulators have displayed promise as biomarkers for the diagnosis, prognosis, and treatment decisions related to HCC. However, the practical application of these biomarkers in HCC necessitates thorough validation across multiple centers in the future. Agents targeting RNA modification are potential therapeutic strategies for HCC. The concurrent administration with the inhibitors of RNA modification may enhance the sensitivity of drugs and offer novel approaches for managing HCC. While a growing number of RNA modifier inhibitors are being developed, the absence of preclinical and clinical studies focusing on targeting RNA modification in HCC presents a significant challenge. Further investigation is crucial to assess the efficacy of these inhibitors in the treatment of HCC.
